# Antimicrobial Resistance in *Escherichia coli* from the Broiler Farm Environment, with Detection of SHV-12-Producing Isolates

**DOI:** 10.3390/antibiotics11040444

**Published:** 2022-03-25

**Authors:** Sandra Martínez-Álvarez, Susana Sanz, Carmen Olarte, Raquel Hidalgo-Sanz, Isabel Carvalho, Rosa Fernández-Fernández, Allelen Campaña-Burguet, Javier Latorre-Fernández, Myriam Zarazaga, Carmen Torres

**Affiliations:** 1 Department of Agriculture and Food, University of La Rioja, 26006 Logroño, Spain; sandra.martinezal@unirioja.es (S.M.-Á.); susana.sanz@unirioja.es (S.S.); carmen.olarte@unirioja.es (C.O.); raquel16196@gmail.com (R.H.-S.); isabelcarvalho93@hotmail.com (I.C.); rosa.fernandez.1995@gmail.com (R.F.-F.); allelencampanaburguet@gmail.com (A.C.-B.); jl471998@gmail.com (J.L.-F.); myriam.zarazaga@unirioja.es (M.Z.); 2 Department of Veterinary Sciences, University of Trás-os-Montes-and Alto Douro, 5000-801 Vila Real, Portugal

**Keywords:** *Escherichia coli*, ESBL, SHV-12, integron, broiler farm, environmental

## Abstract

Antimicrobial resistance is an important One Health challenge that encompasses the human, animal, and environmental fields. A total of 111 *Escherichia coli* isolates previously recovered from manure (*n* = 57) and indoor air (*n* = 54) samples from a broiler farm were analyzed to determine their phenotypes and genotypes of antimicrobial resistance and integron characterization; in addition, plasmid replicon analysis and molecular typing were performed in extended-spectrum-beta-lactamase (ESBL) producer isolates. A multidrug-resistance phenotype was detected in 46.8% of the isolates, and the highest rates of resistance were found for ampicillin, trimethoprim–sulfamethoxazole, and tetracycline (>40%); moreover, 15 isolates (13.5%) showed susceptibility to all tested antibiotics. None of the isolates showed imipenem and/or cefoxitin resistance. Twenty-three of the one hundred and eleven *E. coli* isolates (20.7%) were ESBL producers and carried the *bla*_SHV-12_ gene; one of these isolates was recovered from the air, and the remaining 22 were from manure samples. Most of ESBL-positive isolates carried the *cmlA* (*n* = 23), *tet*(A) (*n* = 19), and *aac*(6′)*-Ib-cr* (*n* = 11) genes. The following genetic lineages were identified among the ESBL-producing isolates (sequence type-phylogroup-clonotype): ST770-E-CH116–552 (*n* = 12), ST117-B2-CH45–97 (*n* = 4), ST68-E-CH26–382/49 (*n* = 3), ST68-E-CH26–49 (*n* = 1), and ST10992-A/B1-CH11–23/41/580 (*n* = 4); the latter two were detected for the first time in the poultry sector. At least two plasmid replicon types were detected in the ESBL-producing *E. coli* isolates, with IncF, IncF1B, IncK, and IncHI1 being the most frequently found. The following antimicrobial resistance genes were identified among the non-ESBL-producing isolates (number of isolates): *bla*_TEM_ (58), *aac*(6′)-Ib-cr (6), *qnrS* (2), *aac*(3)-II (2), *cmlA* (6), *tet*(A)/*tet*(B) (22), and *sul1/2/3* (51). Four different gene-cassette arrays were detected in the variable region of class 1 (*dfrA1-aadA1*, *dfrA12-aadA2*, and *dfrA12-orf-aadA2-cmlA*) and class 2 integrons (*sat2-aadA1-orfX*). This work reveals the worrying presence of antimicrobial-resistant *E. coli* in the broiler farm environment, with ESBL-producing isolates of SHV-12 type being extensively disseminated.

## 1. Introduction

Antimicrobial resistance (AMR) is one of the biggest problems that face health authorities worldwide. For a long time, AMR has been focused on the clinical setting, but nowadays, it is clear that this is a problem that involves humans, animals, and the environment, and AMR bacteria can be disseminated throughout the environment, which requires a One Health approach. The emergence and the spread of AMR are closely related to the extensive use and misuse of antimicrobials in the human and animal fields [1]. In this sense, antimicrobials have been extensively used in food-producing animals, for instance, in the poultry sector, for growth promotion (banned since 2006 in the European Union and other countries but still allowed in many others), as well as for prophylaxis and therapeutics. According to the World Organization for Animal Health (OIE) in the *Report on antimicrobial agents intended for use in animals* published in 2021, 26% of 160 countries analyzed in 2019 were still using antibiotics as growth promoters in animal production [2]. Antimicrobials are frequently used in the poultry sector due to the high mortality rate in the first weeks of life because their immune system is not fully developed, and they are susceptible to bacterial infections [3]. Rapid interventions to reduce the dissemination pathway through environmental pollution or agricultural effluents are indispensable in the fight against AMR [4,5].

*Escherichia coli* is a commensal microorganism of the intestinal microbiota of healthy humans and animals, as well as an important opportunistic pathogen, which may be implicated in many types of infections. Intestinal bacteria (as is the case of *E. coli*) are exposed to the effect of antimicrobial agents used in humans and animals, and the emergence and dissemination of resistance could occur, with AMR bacteria being disseminated in the environment [6,7]. One of the most relevant mechanisms of resistance in *E. coli* that causes great clinical concern is the expression of extended-spectrum beta-lactamases (ESBLs), which confer resistance to penicillins, narrow and broad-spectrum cephalosporins (such as ceftazidime and cefotaxime), and monobactams [8]. Many of the genes encoding ESBLs are hosted on plasmids that facilitate the transfer among bacterial species [9,10,11].

The first report of ESBL-producing *E. coli* isolates detected in food-producing animals was in the poultry sector, specifically in chicken fecal samples in Spain, where SHV-12 and CTX-M-14 producing isolates were found [12]; in this case, they corresponded to two ESBL producers among 120 fecal isolates (1.7%), obtained from chickens during the period 2000–2001. This study was coincident with the increase in ESBL-producing *E. coli* isolates in the hospitals, mostly linked to CTX-M variants and SHV-12, both in Spain and in other European countries [13,14]. In the following years, the reports indicating the detection of ESBL-producing isolates in healthy poultry and other food-producing animals have been increasingly found worldwide [15,16,17]. Nevertheless, the knowledge about the dissemination of AMR genes of clinical relevance (as ESBL-related ones) in the environment of a poultry farm is still scarce.

Multiple genetic mechanisms are implicated in the acquisition and dissemination of AMR. The *E. coli* mobilome includes a variety of mobile and mobilizable genetic elements, including plasmids, transposons, insertion sequences, and integrons (*intI*) [18]. The latter are well known to be involved in the spread of antibiotic resistance, notably among Gram-negative bacteria. Integrons are genetic structures containing AMR genes in their variable region (as gene cassettes) and have been detected in poultry farms in different studies [19,20].

The environmental dissemination of bacteria of *Enterobacteriaceae*, enterococci, and staphylococci groups was analyzed in a broiler farm in a previous study [21]. The aim of the present work is to characterize the *E. coli* isolates obtained from indoor air and manure collected from the previously mentioned study, analyzing AMR phenotypes and genotypes and the presence of integrons, as well as the molecular typing of selected isolates, to evaluate the role of the farm environment in the dissemination of relevant multidrug-resistant *E. coli* isolates.

## 2. Materials and Methods

### 2.1. Bacterial Collection

A total of 111 *E. coli* isolates were obtained in a previous study from environmental samples (manure and air) of a broiler farm [21]. This farm was equipped with automatic feeding and watering systems, and broilers were reared on deep litter and had free access to feed and water. Their production cycle was 45 days, and after completion, manure and litter were cleaned with mobile machinery. Before loading new flocks, the farm was disinfected. Broiler farm samplings were conducted at 15–16 days after chick entry, and temperature and relative humidity inside the building were 27 ± 1 °C and 70%, respectively. Air samples were taken inside the farm by stationary and mechanical sampling methods (Air Ideal), and non-antibiotic supplemented Chromocult coliform agar (CCA, Merck) plates were used for *E. coli* recovery. The manure samples were collected aseptically at the same time and place. Free mobility of the chickens in the enclosure ensured the homogeneity of the litter. Microbiological analysis was performed on 10 g of each litter sample homogenized with 90 mL of sterile peptone water. Serial dilutions of homogenized litter samples were performed and spread on the surface of CCA agar plates. By this procedure, 54 isolates of indoor air and 57 of manure from the farm were obtained. These isolates were identified by MALDITOF-MS (Matrix-Assisted Laser Desorption/Ionization) (Bruker Daltonik, Bremen, Germany) and preserved at −80 °C, and they were included and characterized in the present study.

### 2.2. Antimicrobial Susceptibility Test

Susceptibility testing was performed by the disc diffusion method in Mueller–Hinton agar plates, according to the Clinical Laboratory Standards Institute [22]. The susceptibility of the *E. coli* isolates was tested for 11 antibiotics (abbrev., charge in µg/disk): ampicillin (AMP, 10), amoxicillin/clavulanate (AMC, 20–10), ceftazidime (CAZ, 30), cefotaxime (CTX, 30), cefoxitin (FOX, 30), imipenem (IMP, 10), ciprofloxacin (CIP, 5), gentamicin (GEN, 10), chloramphenicol (CHL, 30), trimethoprim/sulfamethoxazole (SXT, 1.25–23.75), and tetracycline (TET, 30). In addition, ESBL screening was performed by a double-disc synergy test using two 3rd generation cephalosporins (CAZ and CTX) between an AMC disc. In the case of the ESBL-positive isolates, the minimal inhibitory concentration (MIC) for beta-lactams was determined by microdilution using an automatic system (MicroScan^®^ Walkaway system, Beckman Coulter, Inc., Brea, CA, USA). When resistance to at least three families of antimicrobial agents was detected, the isolates were considered multidrug resistant (MDR).

### 2.3. Characterization of Antimicrobial Resistance Genes, Integrons, and Plasmids

DNA extraction from *E. coli* isolates was performed using the boiling method; briefly, one colony of an overnight culture was suspended in 1 mL of MilliQ water, and later, it was boiled for 8 min to break down the cell wall and centrifuged at 12,000 r.p.m. for 2 min to remove the pellet. DNA concentration was checked using the Nanodrop spectrophotometer.

The presence of antimicrobial resistance genes was analyzed by PCR and sequencing: beta-lactams (*bla*_TEM_, *bla*_SHV_, *bla*_CTX-M_, *bla*_CMY_, and *bla*_DHA_), TET (*tet*(A), and *tet*(B)), SXT (*sul1*, *sul2*, *sul3*, *dfrA1* and *dfrA12*), aminoglycosides (*aac*(3)-*II*, and *aadA1/aadA2*), CIP (*aac(6′)-Ib-cr*, *qnrA*, *qnrS*, and *qnrB*), colistin (*mcr-1*), and CHL (*cmlA, floR*, and *catA1*) [23,24]. Furthermore, amino acid substitutions in GyrA and ParC proteins (QRDR region) were analyzed by PCR and sequencing in the CIP-resistant ESBL-producing isolates that lacked CIP resistance acquired genes [15].

In addition, the presence of integrase genes (*int1* and *int2*) of class 1 and class 2 integrons and their variable regions were analyzed by PCR “primer-walking” strategy and subsequent sequencing [11,25].

Analysis of the DNA sequences was accomplished through BLAST software (https://blast.ncbi.nlm.nih.gov/Blast.cgi, accessed on 10 December 2021).

Plasmid incompatibility (Inc) groups were determined by the PCR-based replicon typing (PBRT) [26] in all the ESBL-producing *E. coli* isolates.

### 2.4. Molecular Typing

All the isolates of the collection were assigned to different phylogroups by multiplex PCR assay [27]. Multilocus sequence typing (MLST) of the ESBL-producing isolates was performed by PCR and sequencing of seven housekeeping genes, according to the guidelines available at https://pubmlst.org/bigsdb?db=pubmlst_ecoli_achtman_seqdef (accessed on 10 January 2022), to determine the sequence type (ST) and the clonal complex (CC). Clonotype identification was performed by sequencing of *fumC* and *fimH* genes (CH) [28].

### 2.5. Statistical Analysis

Statistical analysis was carried out in IBM SPSS 26.0 using the chi-square test to compare pairwise differences in resistance rates to each antimicrobial agent between indoor air and manure samples and also among ESBL- and non-ESBL producers. A *p*-value < 0.05 is considered a statistically significant difference. Statistical analysis of the correlation between phenotypic and genotypic resistance was performed by calculating Pearson’s correlation coefficient with R Study (SAS Institute Inc., Cary, NC, USA).

## 3. Results

### 3.1. Antimicrobial Susceptibility Testing

**Figure 1:**
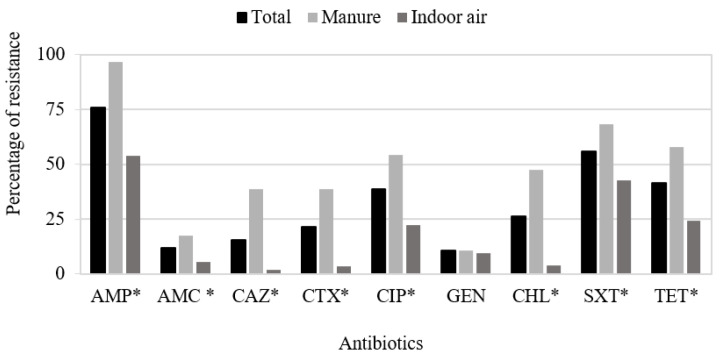
Percentages of antibiotic resistance among the total *E. coli* isolates (*n* = 111) and from those obtained from manure (*n* = 57) and indoor air (*n* = 54). Abbreviations: AMP—ampicillin; AMC—amoxicillin clavulanate; CAZ—ceftazidime; CTX—cefotaxime; CIP—ciprofloxacin; GEN—gentamicin; CHL—chloramphenicol; SXT—trimethoprim/sulfamethoxazole; TET—tetracycline. No resistance for imipenem and cefoxitin was identified among the isolates. * *p*-value < 0.05.

Fifteen of the one hundred and eleven *E. coli* isolates (13.5%) showed susceptibility to all antimicrobial agents tested, and the remaining isolates (86.5%) exhibited resistance to at least one agent, with 52 isolates being considered as MDR (46.8%). High resistance rates were obtained for AMP, SXT, TET, and CIP (75.7–38.7%) and lower rates for CHL, CAZ, CTX, AMC, and GEN (27–10%). No IMP or FOX resistances were found in this collection of isolates. In addition, significant differences were observed between indoor air and manure isolates for AMP, AMC, CAZ, CTX, CIP, CHL, SXT, and TET resistance rates (Figure 1).

Twenty-three of the one hundred and eleven isolates (20.7%) showed a positive ESBL-screening test. The MIC for beta-lactams was determined in the 23 ESBL-positive isolates, and they showed the following MICs (in µg/mL): CTX (>16), CAZ (>8), IMP (≤1), and aztreonam (>4).

**Figure 2:**
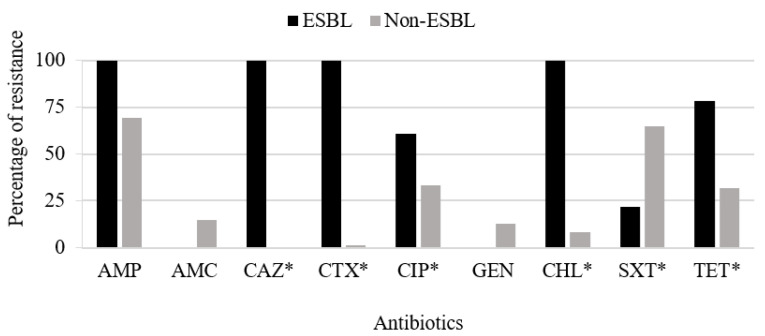
Distribution of resistance phenotypes among ESBL-producing (*n* = 23) and non-ESBL-producing (*n* = 88) *E. coli* isolates of the poultry farm. Abbreviations: AMP—ampicillin; AMC—amoxicillin/clavulanate; CAZ—ceftazidime; CTX—cefotaxime; CIP—ciprofloxacin; GEN—gentamicin; CHL—chloramphenicol; SXT—trimethoprim/sulfamethoxazole; TET—tetracycline. No resistance for imipenem and cefoxitin was identified among the isolates. * *p*-value < 0.05.

Resistance rates for CIP, CHL, and TET were higher among ESBL-positive isolates, but the pattern was different for GEN and SXT. It is important to highlight that significant differences were detected between ESBL- and non-ESBL-producing isolates for CIP, CHL, SXT, and TET, in addition to, as expected, broad-spectrum cephalosporins (Figure 2).

### 3.2. Molecular Characterization and Typing of ESBL-Producing E. coli

A total of 23 of the 111 *E. coli* isolates showed an ESBL phenotype, hosting diverse AMR phenotypes and genotypes, as well as genetic lineages. Most of these isolates were obtained from manure samples, but interestingly, one of the ESBL producers (X2583) was obtained from an indoor air sample. All these isolates carried the gene encoding the beta-lactamase SHV-12, with one of them (X2685) also carrying the gene encoding TEM-1.

Most of the ESBL producers showed CHL and TET resistance and carried the *cmlA* and *tet*(A) genes (with few exceptions). Eleven out of fourteen CIP-resistant isolates harbored the *aac(6′)-Ib-cr* gene. Two additional CIP-resistant isolates presented point mutations in the QRDR region in the *gyrA* and *parC* genes: two amino acid changes were detected in GyrA (S83L, D87N) and one in ParC (S80I). In addition, the *sul1* and *sul3* genes were detected in two and four isolates, respectively.

The 23 SHV-12-producing *E. coli* isolates were typed by MLST, and four different ST were detected: (a) twelve isolates were typed as ST770 and phylogroup-E (52.2%); the isolate from indoor air was included in this group; (b) three isolates were typed as ST68 and phylogroup-E (13%); (c) four isolates were typed as ST117 and phylogroup-B2 (17.4%); and (d) four isolates were typed as ST10992 (17.4%), two of them ascribed to phylogroup-A and the other two to phylogroup-B1. The CH typing identified seven clonotypes, with CH116–552 being the most prevalent, present in 12 ESBL producers associated with ST770. Other prevalent clonotypes were CH45–97 (two ST117 isolates), CH11–41 (two ST10992 isolates), and CH26–382 (two ST68 isolates) (Table 1).

Plasmid characterization revealed the detection of at least two replicon types in each isolate, with IncF, IncF1B, IncK, and IncHI1 being the most frequent types detected, although IncI1, IncP, IncFIC, and IncB/O were also identified (Table 1).

### 3.3. Characterization of Antimicrobial Resistance Genes among Non-ESBL-Producing Isolates

AMR genes were analyzed in the collection of 88 non-ESBL-producing isolates. Fifty-eight of the sixty-one AMP-resistant isolates carried the *bla*_TEM_ gene (95.1%). Several acquired resistance genes, such as *aac(6)-Ib-cr* and *qnrS*, were detected in six and two isolates out of twenty-nine CIP-resistant isolates, respectively. Most of the 28 TET-resistant isolates carried the *tet*(A) and/or *tet*(B) genes, either alone or combined (78.6%). Moreover, 51 of the 57 SXT-resistant isolates (89.5%) carried *sul* genes (*sul1, sul2*, or *sul3*), either alone or associated. The *aac(3)-II* and *cmlA* genes were detected among GEN- and CHL-resistant isolates, respectively (Table 2). All isolates were negative for *mcr-1*, a gene associated with colistin resistance.

The phylogroup typing was performed in all 88 non-ESBL producers with the following distribution (number of isolates): A (*n* = 38), E (*n* = 15), C (*n* = 12), B1 (*n* = 4), F (*n* = 3), B2 (*n* = 1), D (*n* = 1), and Clade I (*n* = 1); thirteen isolates could not be typed.

### 3.4. Correlation between Phenotypic and Genotypic Resistance Profile of E. coli Isolates

**Figure 3:**
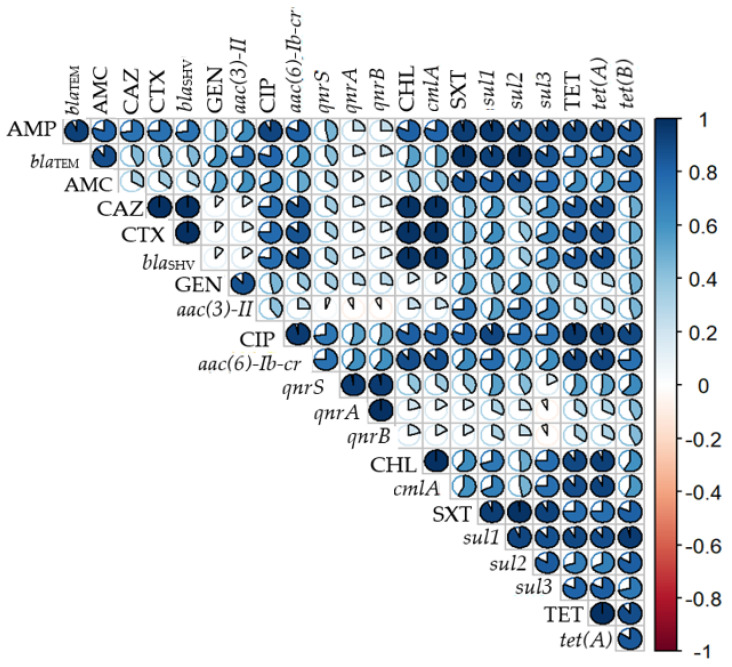
Correlation matrix of phenotypic and genotypic antibiotic resistance of the 111 *E. coli* isolates. Blue is positive correlation and red is negative correlation (1 = positive correlation, 0 = no correlation, and −1 = negative correlation). Size and strength of the color represent numerical value of correlation coefficient. Abbreviations: AMP—ampicillin; AMC—amoxicillin clavulanate; CAZ—ceftazidime; CTX—cefotaxime; CIP—ciprofloxacin; GEN—gentamicin; CHL—chloramphenicol; SXT—trimethoprim/sulfamethoxazole; TET—tetracycline. No resistance for imipenem and cefoxitin was identified among our isolates.

Correlation analysis revealed that the AMR genes analysed correlated positively with their corresponding target antimicrobials. There was a strong positive correlation between AMP and bla_TEM_ (r = 0.93); CAZ-CTX and bla_SHV_ (r = 1); GEN and aac(3)-II (r = 0.87); CIP and aac(6′)-Ib-cr (r = 0.95); SXT and sul2 (r = 0.98), sul1 (r = 0.94), sul3 (r = 0.92); and TE and tet(A) (r = 1). Moreover, a strong positive correlation between AMR genes and antimicrobials of different classes was also observed, for instance, SXT with bla_TEM_ (r = 0.99), CAZ-CTX with cmlA (r = 0.99), CIP with tet(A) (r = 0.95) and sul1 (r = 0.92), CHL with tet(A) (r = 0.93), and TET with cmlA (r = 0.89) and sul1 (r = 0.91). Additionally, a strong positive correlation was detected between antibiotics from different families, among others, AMP with CIP (r = 0.92), CHL (r = 0.81), SXT (r = 0.95), and TET (r = 0.91); CAZ-CTZ with CHL (r = 0.98) and TET (r = 0.84); CIP and TE (r = 0.96); CHL with CIP (r = 0.83) and TET (0.91) (Figure 3).

### 3.5. Characterization of Integrons among STX-Resistant E. coli Isolates

The presence of *intl1* and *intl2* genes was analyzed in the 62 SXT-resistant *E. coli* isolates detected in this study. The *intI1* gene was identified in 26 isolates, *intI2* in 7 isolates, and both genes in 5 additional isolates (Table 2). The variable region of class 1 and/or class 2 integrin’s could be identified in 14 of these isolates, and their gene cassettes arrays are shown in Table 3. In this regard, three types of gene-cassettes arrays (GC) have been detected in the class 1 integrons among the 11 isolates which could be characterized: (1) *dfrA1-aadA1* (*n* = 8); (2) *dfrA12-aadA2* (*n* = 2) linked to *sul1;* and (3) *dfrA12-orfX-aadA2-cmlA* linked to *sul3* (*n* = 1). The variable region of class 2 integrons was identified in five isolates, and the GC found was *sat-aadA1* (Table 3).

## 4. Discussion

The AMR of *E. coli* isolates from the broiler farm environment was analyzed in this study, with a special focus on the characterization of ESBL-producing isolates. Of relevance is the high incidence (20.7%) of ESBL producers detected among the collection of *E. coli* isolates. It is important to note the detection of ESBL-producing *E. coli* isolates of the same genetic lineage and ESBL type in manure (*n* = 11) and indoor air samples (*n* = 1) from the broiler farm, showing dissemination of these resistant bacteria in different niches of the farm.

Among the most relevant results observed concerning the AMR patterns of the antibiotics studied, the highest resistance rates were detected for CHL, TET, and CIP (in ESBL-producing isolates, in addition to β-lactams), and for AMP, CIP, TET, and SXT (in non-ESBL-producing isolates) coinciding closely with the results reported by EFSA [1]. This could be associated with the high use of these antimicrobials in the poultry sector. The statistical analysis revealed a close correlation between antibiotics from different families, which might be explained by the location of the implicated resistance genes in the same genetic structures that could be co-selected.

In the present study, all the 23 ESBL producers carried the gene encoding the SHV-12 enzyme. This ESBL is the most widespread variant in European countries in the poultry sector [9,16,29,30] and has also been frequently detected in Spanish hospitals [14]. Nevertheless, other ESBL variants (TEM-52, CTX-M-14, CTX-M-1, CTX-M-15, CTX-M-32, and CTX-M-9, among others) and pAmpC enzymes (CMY-2) have also been reported in the poultry sector, including slaughterhouse wastewater [9,15,31,32,33,34]. In Spanish hospitals, several studies have shown that CTX-M-15 and CTX-M-14 were the predominant ESBL enzymes [35,36]. Furthermore, ESBL-producing *E. coli* isolates have been frequently detected in fecal samples from healthy humans in Spain with rates of 7–16% [24,29]. The possibility of the food chain being involved in the acquisition of ESBL-producing isolates has been postulated [37].

ESBL genes could be transferred via plasmids between bacteria of diverse origins. The F, FIB, K, and HI1 plasmids were frequently detected among the SHV-12-producing isolates in poultry in other studies [38,39]. Moreover, IncI1 plasmids are also found associated with *bla*_SHV-12_ in the human and animal environment [17,40] and even in food isolates [10,30], where IncK and IncX3 replicons were also detected, in lower proportions. The IncF, IncF1B, IncK, IncHI1, and IncI1 replicon types have also been detected among our ESBL-producing isolates. Additional research is necessary to analyze in greater depth the genetic information associated with the ESBL and/or MDR phenotypes.

Four different genetic lineages (ST-phylogroup) were detected among SHV-12-producing isolates in this study: ST770-E (predominant, 52% of isolates), ST117-B2 (17.4%), ST10992-A/B1 (17.4%), and ST68-E (13%).

The ST770 lineage has been previously reported in CTX-M-1- or CMY-2-producing *E. coli* from poultry or chicken meat in different European countries [39,41] and associated with ESBL-negative avian pathogenic *E. coli* (APEC) [42]. Moreover, ST770 has also been detected in CTX-M-14- and CTX-M-2-producing *E. coli* isolates implicated in urinary tract infections [43]. Interestingly, this lineage has also been reported in ESBL producers from litter and poultry farm air (SHV and TEM-types), which reinforces the importance of cleaning and disinfection protocol to remove the microbial load before introducing the litter into the farms [44], according to the “all-in-all-out” strategy to avoid the dissemination of MDR bacteria. Furthermore, this lineage has also been detected in isolates carrying very relevant resistance genes, such as *mcr-1* (colistin resistance), in that case in pets [45]. Data collected from studies in several countries show an increased association of the ST770 lineage with ESBL- and pAmpC-producing *E. coli* isolates.

Three of the lineages detected in this study (ST68, ST770, and ST117) have been detected in SHV-12, CTX-M-1, CTX-M-14, and/or CTX-M-15-positive *E. coli* isolates from food-producing animals [17]. In other origins, such as wildlife and companion animals, these three genetic lineages were found associated with the production of CMY-2 [46,47] and ST117 with the expression of CTX-M-1. [48]. In addition, ST68 has been reported in carbapenemase-positive isolates (OXA-48) [49]. Nevertheless, to our knowledge, the lineage ST68 has not been detected in Europe in SHV-12-producer isolates of poultry origin.

The ST117 is the most widespread genetic lineage in the poultry sector, being also the most frequent in our study. This lineage has been detected in SHV-12 producers from poultry slaughterhouse wastewater in Germany [34], and its high prevalence continues in the broiler production pyramid [50]. This lineage has also been detected in avian farming in ESBL-positive isolates carrying genes of the CTX-M family (CTX-M-1, CTX-M-14, CTX-M-15) or in CMY-2 producers [51]. In this sense, ST117 is considered a reservoir for ESBL and pAmpC encoding genes in poultry and is also closely associated with APEC isolates [52,53].

The unusual ST10992 lineage was firstly reported in this study, to our knowledge, in the poultry farm environment, among SHV-12-producing isolates.

Phylogenetic grouping provides epidemiological and ecological information related to the virulence capacity of *E. coli* isolates. Several studies revealed that phylogroups B2, D, E, and F are strongly associated with extraintestinal infections in humans (EXPEC strains), whereas A and B1 are generally associated with commensal bacteria in humans and animals, including those ESBL producers [43,51]. In this sense, phylogroups A, B1, and F have been frequently found among ESBL-producing *E. coli* isolates from poultry farms in different European studies, with phylogroups B2 and D scarcely detected [50,54]. Studies from the poultry environment in Africa reveal the predominance of phylogroups A and D [55]. In our study, the presence of A and B1 was evident among ESBL-producing isolates, although phylogroups E and B2 were also frequently detected.

Integrons are mobilizable genetic elements whose study in recent years has gained relevance due to their role in the acquisition and dissemination of AMR. Four types of gene cassette arrays were detected among the eleven characterized isolates in our study, associated to class 1 (*dfrA1-aadA1*, *dfA12-aadA2,* and *dfrA12-orfX-aadA2-cmlA*) and class 2 integrons (*sat2-aadA1*). They carried resistance genes to old antibiotics, such as trimethoprim (*dfrA1*, *dfrA12*), streptomycin (*aadA1*, *aadA12*), chloramphenicol (*cmlA*), or streptothricin (*sat2*), whose use in animals or humans could contribute to the selection of integron-positive isolates. The presence of integrons in poultry *E. coli* isolates containing the classical *dfrA1-aadA1* structure has been previously reported [52,56].

Potential sources of AMR in poultry production consist of air, dust, soil, feed, and rodents or other animals. From these sources, AMR bacteria and genes can be transmitted to humans through the food chain or indirectly by the environment, posing a threat to public health [1]. Several studies have shown the role of air as a disseminating vehicle and the ability to persist both inside and outside a farm, and poultry manure is considered to be the main vector for the spread of AMR bacteria [55].

## 5. Conclusions

ESBL-producing isolates frequently contaminate the poultry farm environment, and these resistant bacteria could be present in the farm air. In our case, SHV-12 was the only ESBL type detected, which has been observed in different *E. coli* clones, some of them very frequent in the poultry sector in both ESBL-producing and APEC isolates. Furthermore, the detection of the same genetic lineage in both poultry manure and airborne isolates provides further evidence of the transmissibility of these resistant bacteria and their ability to survive and adapt to any niche. Close surveillance on the dissemination of ESBL-producing isolates in the farm environment should be performed to avoid the dissemination of these isolates through the food chain or indirectly through the air or dust, among others.

This study shows that both manure and airborne dust particles are important sources of resistant bacteria and AMR genes due to the high survival of these resistant microorganisms, making it necessary to apply appropriate cleaning protocols to reduce and avoid as much as possible the survival of bacteria. Based on these results, it is suggested that broiler farms could be considered as important vehicles for the transmission of multidrug-resistant bacteria favoring their spread into the natural environment and in turn posing a risk to public health.

AMR is probably the clearest example of a global and growing problem that requires solutions to deal with the “One Health” approach. Coordinated and precise actions are needed to reduce its impact now and in the future, as well as measures to ensure the economic viability of the poultry sector together with public health assurance.

## Figures and Tables

**Table 1:** Determinants of resistance and molecular typing of the 23 ESBL-producing *E. coli* isolates.

*E. coli* Isolate	Origin	Resistance Phenotype ^a^	Resistance Genotype	Phylogroup, Sequence Type (MLST), and Clonotype	Replicon Type
X2583	Airborne	AMP, CAZ, CTX, CHL, CIP, TET	*bla*_SHV-12_, *cmlA*, *tet*(A), *aac(6′)-Ib-cr*	E-ST770-CH116–552	IncF, IncK, IncB/O
X2685	Manure	AMP, CAZ, CTX, CHL, CIP, TET	*bla*_SHV-12_, *bla*_TEM-1_, *cmlA*, *tet*(A), *aac(6′)-Ib-cr*	E-ST770-CH116–552	IncFIB, IncF, IncK
X2636	Manure	AMP, CAZ, CTX, CHL, CIP, TET	*bla*_SHV-12_, *cmlA*, *tet*(A), *aac(6′)-Ib-cr*	E-ST770-CH116–552	IncFIB, IncF, IncK
X2630	Manure	AMP, CAZ, CTX, CHL, CIP, TET	*bla*_SHV-12_, *cmlA*, *tet*(A), *aac(6′)-Ib-cr*	E-ST770-CH116–552	IncFIB, IncF, IncB/O
X2686	Manure	AMP, CAZ, CTX, CHL, CIP, TET	*bla*_SHV-12_, *cmlA*, *tet*(A), *aac(6′)-Ib-cr*	E-ST770-CH116–552	IncF, IncK
X2635	Manure	AMP, CAZ, CTX, CHL, CIP, TET	*bla*_SHV-12_, *cmlA*, *tet*(A), *aac(6′)-Ib-cr*	E-ST770-CH116–552	IncFIB, IncF, IncK
X2637	Manure	AMP, CAZ, CTX, CHL, CIP, TET	*bla*_SHV-12_, *cmlA*, *tet*(A), *aac(6′)-Ib-cr*	E-ST770-CH116–552	IncFIB, IncF, IncK
X2639	Manure	AMP, CAZ, CTX, CHL, CIP, TET	*bla*_SHV-12_, *cmlA*, *tet*(A), *aac(6′)-Ib-cr*	E-ST770-CH116–552	IncI1, IncFIB, IncF, IncK
X2633	Manure	AMP, CAZ, CTX, CHL, CIP, TET	*bla*_SHV-12_, *cmlA*, *tet*(A), *aac(6′)-Ib-cr*	E-ST770-CH116–552	IncFIB, IncF, IncK
X2683	Manure	AMP, CAZ, CTX, CHL, CIP, TET	*bla*_SHV-12_, *cmlA*, *tet*(A), *aac(6′)-Ib-cr*	E-ST770-CH116–552	IncF, IncK
X2684	Manure	AMP, CAZ, CTX, CHL, CIP, TET	*bla*_SHV-12_, *cmlA*, *tet*(A), *aac(6′)-Ib-cr*	E-ST770-CH116–552	IncFIB, IncF, IncK
X2638	Manure	AMP, CAZ, CTX, CHL, CIP ^b^, TET	*bla*_SHV-12_, *cmlA*, *tet*(A)	E-ST770-CH116–552	IncFIB, IncF, IncK
X2632	Manure	AMP, CAZ, CTX, CHL, CIP ^b^, TET	*bla*_SHV-12_, *cmlA*, *tet*(A)	E-ST68-CH26–382	IncFIB, IncF
X2634	Manure	AMP, CAZ, CTX, CHL	*bla*_SHV-12_, *cmlA*, *tet*(A)	E-ST68-CH26–382	IncFIB, IncF
X2682	Manure	AMP, CAZ, CTX, CHL, CIP	*bla*_SHV-12_, *cmlA*	E-ST68-CH26–49	IncI1, IncP, IncF
X2631	Manure	AMP, CAZ, CTX, CHL, SXT, TET	*bla*_SHV-12_, *cmlA*, *tet*(A), *sul1*, *dfrA1*, *aadA1*	B2-ST117-CH45–97	IncFIB, IncF, IncB/O
X2640	Manure	AMP, CAZ, CTX, CHL	*bla*_SHV-12_, *cmlA*	B2-ST117- CH45–97	IncFIB, IncF, IncK
X2641	Manure	AMP, CAZ, CTX, CHL	*bla*_SHV-12_, *cmlA*	B2-ST117-CH45–97	IncFIB, IncF, IncK
X2642	Manure	AMP, CAZ, CTX, CHL	*bla*_SHV-12_, *cmlA*	B2-ST117-CH45–97	IncFIB, IncF, IncK
X2643	Manure	AMP, CAZ, CTX, CHL, SXT, TET	*bla*_SHV-12_, *cmlA*, *tet*(A), *sul1*, *sul3*, *dfrA1*, *aadA1*	A-ST10992-CH11–23	IncFIB, IncF
X2644	Manure	AMP, CAZ, CTX, CHL, SXT, TET	*bla*_SHV-12_, *cmlA*, *tet*(A), *sul3*	A-ST10992-CH11–41	IncFIB
X2646	Manure	AMP, CAZ, CTX, CHL, SXT, TET	*bla*_SHV-12_, *cmlA*, *tet*(A), *sul3*	B1-ST10992-CH11–41	IncP, IncI1
X2645	Manure	AMP, CAZ, CTX, CHL, SXT, TET	*bla*_SHV-12_, *cmlA*, *tet*(A), *sul3*	B1-ST10992-CH11–580	IncP, IncK

^a^ AMP—ampicillin; CAZ—ceftazidime; CTX—cefotaxime; CIP—ciprofloxacin; GEN—gentamicin; CHL—chloramphenicol; SXT—trimethoprim/sulfamethoxazole; TET—tetracycline. ^b^ These isolates showed the amino acid changes S83L-D87N and S80I in GyrA and ParC proteins, respectively.

**Table 2:** Antimicrobial resistance genes detected among the 88 non-ESBL-producing *E. coli* isolates in relation to their phenotype of resistance.

Antibiotic	Number of Resistant Isolates	Resistance Genes Detected (Number of Isolates)	Integrase of Class 1/2 Integrons (Number of Isolates)
Ampicillin	61	*bla*_TEM_ (58)	*-*
Ciprofloxacin	29	*aac6′-Ib-cr* (6), *qnrS* (2)	*-*
Gentamicin	11	*aac(3)-II* (2)	*-*
Chloramphenicol	7	*cmlA* (6)	*-*
SXT ^a^	57	*sul1* (2), *sul2* (31), *sul3* (7), *sul1 + sul2* (9), *sul2 + sul3* (2)	*int1* (26), *int2* (7), *int1 + int2* (5)
Tetracycline	28	*tet*(A) (12), *tet*(B) (3), *tet*(A) + *tet*(B) (7)	

^a^ SXT—trimethoprim/sulfamethoxazole.

**Table 3:** Integron characterization in representative SXT-resistant *E. coli* isolates.

*E. coli* Isolate	Origin	Resistance Phenotype ^a^	ESBL Phenotype ^b^	Resistance Genotype	Phylogroup (MLST)	Class 1 Integron		Class 2 Integron	
						*intl1*/3′CS	VR ^c^	*intl2*	VR ^c^
X2631	Manure	AMP, CAZ, CTX, CHL, SXT, TET	+	*bla*_SHV-12_, *cmlA*, *tet*(A)	B2 (ST117)	+/+	*dfrA1-aadA1*	−	
X2643	Manure	AMP, CAZ, CTX, CHL, SXT, TET	+	*bla*_SHV-12_, *cmlA*, *tet*(A), *sul3*	A (ST10992)	+/+	*dfrA1-aadA1*	−	
X2576	Airborne	AMP, SXT, TET	−	*bla*_TEM-1_, *tet*(A), *sul2*	A (ST5766)	+/+	*dfrA1-aadA1*	−	
X2671	Manure	AMP, CIP, SXT, TET	−	*bla*_TEM-1_, *tet*(A), *sul2*	D (ST69)	+/+	*dfrA1-aadA1*	−	
X2579	Airborne	AMP, CIP, SXT, TET	−	*bla*_TEM-1_, *tet*(A), *sul2*	A	+/+	*dfrA1-aadA1*	−	
X2580	Airborne	AMP, CIP, SXT, TET	−	*bla*_TEM-1_, *tet*(A), *sul2*	E	+/+	*dfrA1-aadA1*	−	
X2675	Manure	AMP, AMC, CIP, SXT, TET	−	*bla*_TEM-1_, *tet*(A), *sul2*	A	+/+	*dfrA1-aadA1*	−	
X2679	Manure	AMP, SXT	−	*bla*_TEM-1_, *sul2*	A	+/+	*dfrA1-aadA1*	−	
X2674	Manure	AMP, AMC, CIP, SXT, TET	−	*bla*_TEM-1_, *tet*(B), *sul2*	A	+/+	*dfrA12-aadA2*	+	*sat2-aadA1*
X2680	Manure	AMP, CHL, CIP, SXT, TET	−	*tet*(A), *aac(6′)-Ib-cr*, *qnrS*	E	+/+	*dfrA12-aadA2*	−	
X2681	Manure	AMP, CHL, CIP, SXT, TET	−	*tet*(B), *cmlA*, *aac(6′)-Ib-cr*, *sul2*, *qnrS*	A	+/+	*dfrA12-orfX-aadA2-cmlA*	−	
X2665	Manure	AMP, AMC, TET, SXT, CHL	−	*bla*_TEM-1_, *cmlA*, *tet*(A), *sul2*, *sul3*	C	+/−		+	*sat2-aadA1*
X2662	Manure	AMP, AMC, TET, SXT, CHL	−	*bla*_TEM-1_, *cmlA*, *sul3*	A	+/−		+	*sat2-aadA1*
X2658	Manure	AMP, TET, SXT, CHL	−	*bla*_TEM-1_, *tet*(A), *sul3*, *cmlA*	A	+/−		+	*sat2-aadA1*
X2666	Manure	AMP, TET, SXT, CHL		*bla*_TEM-1_, *cmlA*, *tet*(A), *sul3*	C	+/−		+	*sat2-aadA1*

^a^ AMP—ampicillin; AMC—amoxicillin clavulanate; CAZ—ceftazidime; CTX—cefotaxime; CIP—ciprofloxacin; GEN—gentamicin; CHL—chloramphenicol; SXT—trimethoprim/sulfamethoxazole; TET—tetracycline. ^b^ Phenotype positive (+) or negative (−). ^c^ VR—variable region.
